# The use of circulating miRNAs for the diagnosis, prognosis, and personalized treatment of MASLD

**DOI:** 10.1007/s13105-025-01110-w

**Published:** 2025-07-16

**Authors:** Ana Luz Tobaruela-Resola, Fermín I. Milagro, Paola Mogna-Pelaez, María Jesús Moreno-Aliaga, Itziar Abete, María Ángeles Zulet

**Affiliations:** 1 Department of Nutrition, Food Sciences and Physiology, Centre for Nutrition Research, Faculty of Pharmacy and Nutrition, University of Navarra, Pamplona, 31008 Spain; 2 Navarra Institute for Health Research (IdiSNA), Pamplona, 31008 Spain; 3 Centro de Investigación Biomédica en Red de Fisiopatología de la Obesidad y Nutrición (CIBERobn), Instituto de Salud Carlos III, Madrid, 28029 Spain

**Keywords:** Circulating MiRNAs, MASLD, Minimally invasive biomarkers, Diagnosis, Prognosis, Personalized treatment

## Abstract

Introduction: Metabolic dysfunction-associated steatotic liver disease (MASLD), formerly NAFLD, includes a range of conditions from steatosis to hepatocellular carcinoma and poses a significant health and economic burden. Circulating microRNAs (miRNAs) are key regulators of metabolic and inflammatory pathways involved in MASLD. However, their clinical utility as non-invasive biomarkers remain unclear. This review aims to clarify their diagnostic, prognostic, and therapeutic potential, addressing current gaps in the literature. Methods: Following PRISMA guidelines, we conducted a systematic review of 1149 studies from the PubMed and Scopus databases up to 2024, focused on circulating miRNAs in MASLD. Results: The most frequently studied miRNAs included miR-122 (35.56% of studies), miR-21 (18.89%), miR-34 (14.44%), and miR-192-5p (13.33%). Diagnostic accuracy varied among miRNAs, with miR-200 and miR-298 demonstrating AUROCs of 0.96 and 0.98, respectively, for MASLD detection. In MASH, miR-200, miR-298, and miR-342 exhibited near-perfect AUROCs of 0.99, while miR-122 showed values between 0.81 and 1.0. For HCC, miR-214 achieved an AUROC of 0.88, and miR-34a ranged from 0.73 to 0.76. Several miRNA panels demonstrated high diagnostic accuracy, with AUROCs up to 0.99, particularly in distinguishing HCC from other liver conditions. Prognostically, elevated miR-122 levels correlated with disease severity and fibrosis progression, while miR-21 and miR-223 were linked to obesity-associated MASH. Therapeutic interventions, including surgery, dietary modifications, and supplementation, were found to modulate miRNA profiles. Conclusions: MiRNAs exhibit strong potential as minimally invasive biomarkers for MASLD, contributing to improved diagnosis, prognosis, and therapeutic decision-making. Their stability and role in personalized medicine underscore their clinical relevance.

**Supplementary Information:**

The online version contains supplementary material available at 10.1007/s13105-025-01110-w.

## Key points

Circulating miRNAs show strong diagnostic value across MASLD and related stages.

MiR-122 is the most studied miRNA and correlates with fibrosis and MASLD severity.

Lifestyle and surgical treatments induce relevant changes in miRNA profiles.

Specific miRNA panels exhibit high accuracy in distinguishing MASLD subtypes.

**Supplementary Information:**

The online version contains supplementary material available at 10.1007/s13105-025-01110-w.

## Introduction

Metabolic dysfunction-associated steatotic liver disease (MASLD), previously called non-alcoholic fatty liver disease (NAFLD), includes a range of liver conditions, which starts with steatotic liver disease (SLD), defined by ≥ 5% liver fat, progresses to metabolic dysfunction-associated steatohepatitis (MASH), with or without fibrosis, and can advance to cirrhosis, liver failure, and hepatocellular carcinoma (HCC) [1].

MASLD affects 30% of the population and this rate is anticipated to rise even more [2]. The prevalence of MASLD ranges from 24 to 48% in the North American population, with similar rates observed in South America, estimated at about 30%. In Europe, the average prevalence of MASLD is reported to be between 23% and 33%. However, there is variation among European countries, with Western European nations experiencing the highest rates of 25–30%, while Eastern countries report lower rates of around 20%. Although there is limited research available in Africa, the estimated prevalence of MASLD is approximately 20% [3]. Furthermore, a meta-analysis shows that the prevalence of MASLD in Asia is roughly 30%, whereas in Australia, it stands at about 16% [4].

Metabolic dysfunction-associated steatotic liver disease (MASLD) is associated with a mortality rate ranging from 0.5 to 9%, primarily due to liver-related complications [5]. Both MASLD and its more advanced form, metabolic dysfunction-associated steatohepatitis (MASH), represent an increasing burden on patients, healthcare systems, and the economy, especially in advanced stages with severe fibrosis [6].

One of the main challenges in addressing these conditions is their underdiagnosis, due to several factors: low disease awareness and understanding of its potential impact, lack of standardized diagnostic criteria, difficulties faced by both patients and healthcare professionals in identifying symptoms and tracing their origin, and the absence of approved pharmacological treatments. As a result, MASLD is often identified incidentally, following abnormal blood test results or liver imaging findings. In this context, raising awareness and deepening the understanding of MASLD is essential to promote early diagnosis, reduce long-term healthcare costs, and improve patients’ quality of life [6]. Effectively tackling MASLD as a public health issue requires personalized prevention and treatment strategies, along with reliable tools to assess treatment response in routine clinical practice [7].

Currently, accurate diagnosis and staging of MASLD often require an invasive liver biopsy, a procedure that carries certain risks. For this reason, research has increasingly focused on developing non-invasive biomarkers to detect and monitor the disease more safely and accessibly [8].

Biomarker research at the clinical level helps understand disease phases, including prediction, diagnosis, and prognosis. Biomarkers are categorized into diagnostic, monitoring, predictive, and prognostic types. Diagnostic biomarkers confirm disease presence, aiming for high sensitivity and specificity. Monitoring biomarkers track disease status and treatment response. Predictive biomarkers identify individuals likely to experience specific effects and are used in trials and patient care. Prognostic biomarkers estimate the likelihood of future clinical events in patients [9].

Although the development of MASLD involves a complex interplay of factors, current evidence suggests its association with the gut microbiome, bile acids, immunity, adipokines, oxidative stress, and genetic and epigenetic factors [10].

MicroRNAs (miRNAs), a class of small non-coding RNAs, play a crucial role in regulating gene expression. MiRNAs serve as valuable markers for the diagnosis, prognosis, and treatment of various human diseases as well as different types of cancer [11]. The dysregulation of different miRNA profiles alters the stability of biological systems by controlling vital biological processes such as metabolism, cellular growth, differentiation, stress responses, and tissue remodeling [12]. Interestingly, miRNAs have been demonstrated to not only regulate intracellular gene expression but also exert intercellular regulatory roles. They are released via extracellular vesicle (EVs) and can impact neighboring or distant cells [13].

There is substantial evidence suggesting that miRNAs are involved in the pathophysiological mechanisms underlying the development of MASLD [14]. Some studies indicate that several miRNA expressions can reduce triglyceride (TG) accumulation and inhibit adipocyte differentiation, indirectly affecting lipid metabolism, including TG binding, cholesterol transport, and fatty acid synthesis and oxidation [15]. In patients with MASLD/MASH, HCC development can be influenced by some miRNAs, which may also be used as diagnostic markers when found in exosomes derived from HCC [16]. Understanding the molecular mechanisms that cause the disease will help in creating effective treatments and might offer understanding on how to prevent these conditions in the future [17], since their role in regulating post-transcriptional modifications and gene expression, along with their remarkable stability, miRNAs are increasingly used for disease detection, monitoring progression, and guiding therapy [18].

There is a gap in the knowledge of the role of miRNAs in MASLD, and particularly in its diagnosis, prognosis and treatment. The current information is fragmented and there is a need to compile and unify the existing information to clarify and understand the contribution of miRNAs in the prevention and pathophysiology of the disease. More importantly, there is a need to know the possible use of circulating miRNAs as non-invasive biomarkers that can help in the management of MASLD, particularly, as early biomarkers or a new tool for personalized treatment.

Therefore, in this review, our emphasis is on exploring how miRNAs contribute to these mechanisms involved in MASLD. Additionally, we discuss the potential of circulating miRNAs as minimally invasive tools for the diagnosis, prognosis, and personalized treatment of MASLD.

## Methods

The present study adheres to the guidelines established by the Preferred Reporting Items for Systematic Reviews and Meta-Analyses (PRISMA) to explore the use of circulating miRNAs for diagnosis, prognosis, and personalized treatment of MASLD.

### Eligibility criteria

We performed a literature review encompassing all types of studies that offered data on variations in miRNA expression between MASLD patients and controls in the different stages of the disease. The study selection process consisted of two stages establishing inclusion and exclusion criteria to select the relevant studies. In the first stage, the titles and abstracts of all retrieved articles were examined, considering the following criteria: (1) studies in humans with MASLD, (2) measurement of miRNAs in plasma/serum and/or liver, (3) original research (excluding reviews, conference abstracts, proceedings, or case reports), (4) written in English, (5) no duplicates, (6) no in silico evaluation and bioinformatics analyses (7) published in the last 10 years (8) and adult population (9). In the second stage, the articles selected in the first stage were read in their entirety to assess their final eligibility and for data collection. The primary metrics used to assess differentiating potential included the area under the receiver operating characteristic (ROC) curve (AUC), fold change, and *p*-values, with statistical significance set at *p* < 0.05.

### Data source and search strategy

All pertinent articles regarding the use of miRNA for diagnosing, prognosing, or treating MASLD that were accessible in the PubMed and Scopus electronic databases until 2024, were reviewed. The search strategy used keywords associated with the following topics: (1) miRNA expression; (2) biomarkers; (3) liver disease. The following strategies were used: “miRNAs OR microRNAs OR microRNA” AND “NAFLD OR MASLD” AND “prognostic OR prognosis OR Prediction”; “miRNAs OR microRNAs OR microRNA” AND “NAFLD OR MASLD” AND “diagnostic OR diagnosis OR detection”; “miRNAs OR microRNAs OR microRNA” AND “NAFLD OR MASLD” AND “treatment OR Therapy OR management”.

### Data collection

Data from the included studies was extracted and organized into an Excel database. Key fields recorded included study identifiers (title, authors, name of the study), country, sample size, stage of the disease, biological source, type of search, sex, and age, gene pathways related to MASLD or biological processes, predictive capacity for the disease and miRNA expression differences between groups.

### Generative IA and IA-assisted technologies in the writing process

During the preparation of this work the author used OpenAI in order to enhance readability and language of the manuscript. After using this tool, the author reviewed and edited the content as needed and take full responsibility for the content of the publication.

## Results

### Study characteristics

**Fig. 1:**
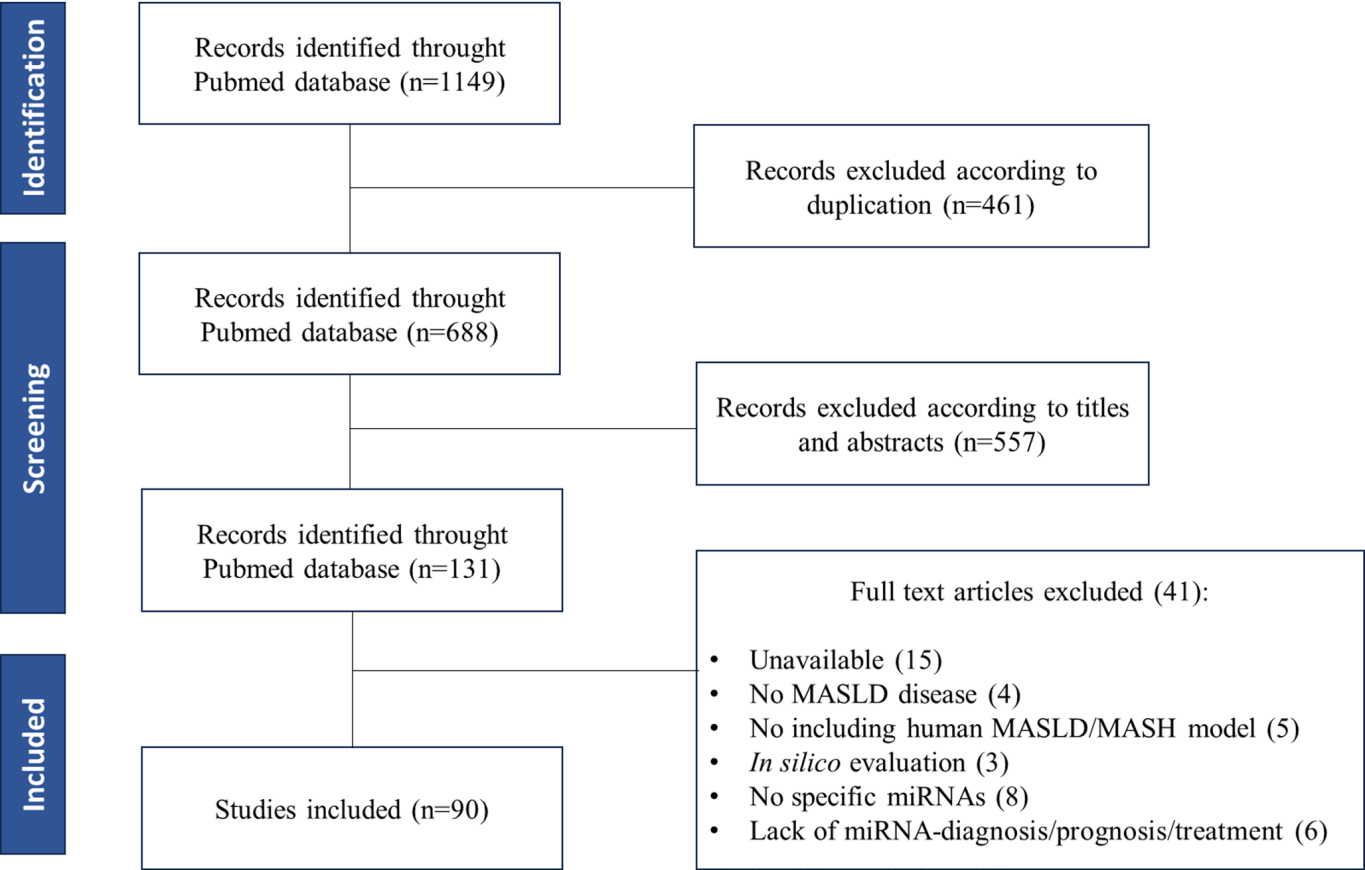
Flowchart of the systematic review Abbreviations: MASLD, Metabolic Dysfunction-Associated Steatotic Liver Disease; MASH, Metabolic Dysfunction-Associated Steatohepatitis; miRNA, microRNA

The systematic literature review resulted in a total of 1149 articles. Of these, 235 corresponded to the first search strategy, 473 to the second, and 441 to the third. After a preliminary screening 461 were removed based on duplication, 557 removed after title abstract screening and the remainder 131 articles were reviewed for both full texts. From these, 41 articles were excluded because they were: unavailable (15), no MASLD disease (4), no including human MASLD/MASH model (5), in silico evaluation (3), no specific miRNAs (8) and lack of miRNA-diagnosis/prognosis/treatment (6). A total of 90 articles satisfied the inclusion criteria and were added to this review. A flowchart of the selection process is shown in Fig. 1. The detailed attributes of these articles are described in Supplementary Table 1 [19–108].

The average age of the participants was 50.06 years. A total of 9553 participants were included, of whom 4718 were male, 4308 were female and 527 had gender unknown.

**Fig. 2:**
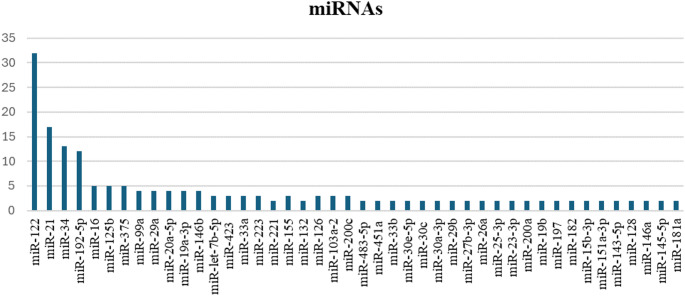
MiRNAs mentioned in all studies of this review. Abbreviations: miRNA, microRNA

A total of 90 studies were included in this systematic review, examining the role of various miRNAs in the pathophysiology of liver disease. The most frequently mentioned miRNAs across the reviewed studies (Fig. 2) were miR-122, which appeared in 35.56% of studies (32), followed by miR-21 in 18.89% (17), miR-34 in 14.44% (13) and miR-192 in 13.33% (12). Other frequently mentioned miRNAs are miR-375, miR125b and miR-16, all of them in 5.56% (5) and miR-19a, miR-29a, miR-99a, miR146b and miR-20a-5p, all in 4.44% of the studies (4). Additionally, some miRNAs were reported less frequently, with appearances in fewer than 4% of studies. These include miR-155, miR-132, miR-200c, miR-223, miR-103a-2, miR-33a, miR-126, miR-let-7b-5p and miR-423, each appearing in only 3.33% of studies (3), among others.

**Fig. 3:**
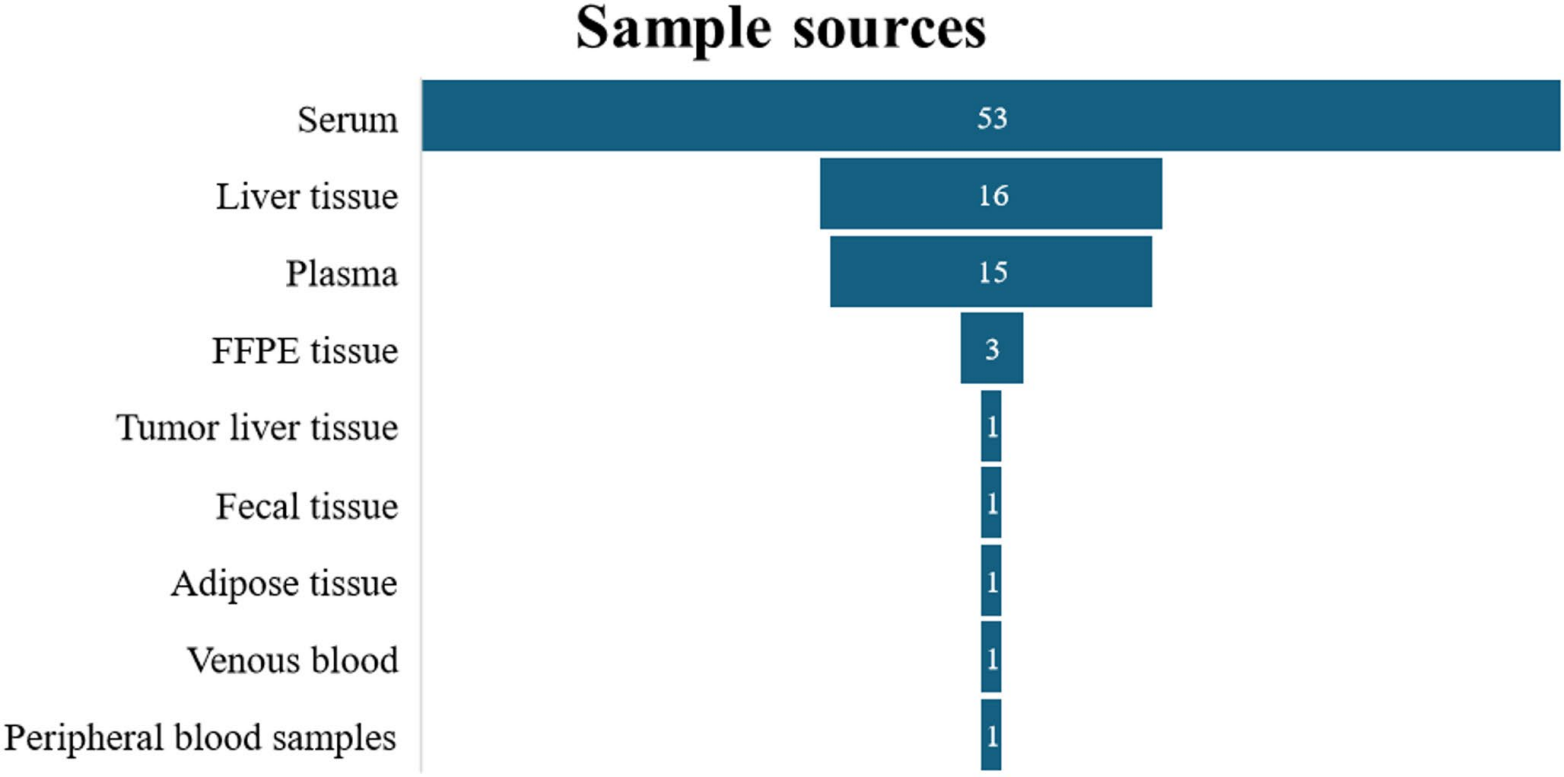
Distribution of sample sources and their frequency in the systematic review. Abbreviations: FFPE, Formalin-Fixed Paraffin-Embedded

All miRNAs were extracted from various sample types. The distribution and frequency of the sample types across the included studies are summarized in Fig. 3.

The most common sources of samples were serum (53 studies) followed by liver tissues (16 studies) and plasma (15 studies). Other frequently sources of sample were Formalin-Fixed Paraffin-Embedded (FFPE) tissue (3 studies). Less frequently, studies used samples such as adipose tissue, tumor liver tissue, fecal samples, venous blood, and peripheral blood samples with only one study each analyzing these sources. This information highlights the predominant use of blood-derived samples (serum and plasma) in miRNA research within the reviewed literature.

**Fig. 4:**
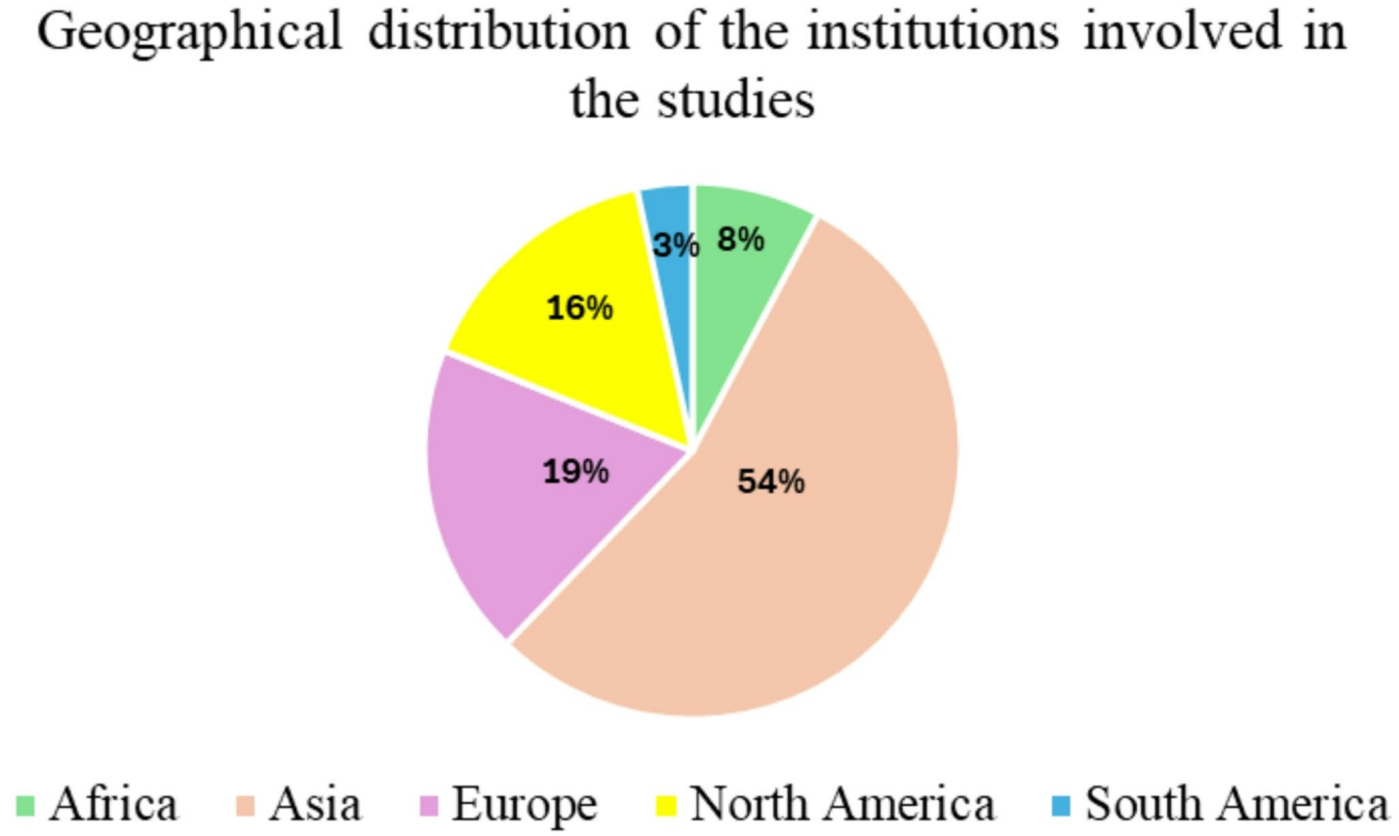
Geographical distribution of the institutions involved in the studies about miRNAs

This distribution highlights the prevalence of specific miRNAs, particularly miR-122, as potential biomarkers in liver disease associated with metabolic syndrome, while other miRNAs were less frequently represented but remain relevant in more specific contexts. A summary of the geographical distribution of the institutions involved in the studies is presented in Fig. 4.

This reveals that most of the research originated from Asia (49 studies), followed by North America (14 studies), Europe (17 studies), Africa (7 studies) and South America (3).

### Diagnostic accuracy of Circulating MicroRNAs in MASLD subjects

Circulating miRNAs demonstrated varying diagnostic accuracies in distinguishing MASLD subtypes, including MASL, MASH and advanced liver conditions. Their performance, measured through the area under the receiver operating characteristic curve (AUROC), underscores their potential as minimally invasive diagnostic tools.

For the early stage of the disease, MASLD (Table 1), miR-122 demonstrated a wide range of diagnostic accuracy, with AUROCs between 0.67 and 0.85 across multiple studies. Other miRNAs, such as miR-200 and miR-298, exhibited outstanding diagnostic performance with AUROCs of 0.96 and 0.98, respectively. Similarly, miR-342 reached an AUROC of 0.94, while miR-6888-5p and miR-193a-5p showed strong performances with AUROCs of 0.91 and 0.92, respectively. MiR-135a and miR146a have also a high accuracy with AUROCs of 0.84 and 0.80, respectively. Additional miRNAs like miR-34a and miR-192 displayed moderate accuracy, with AUROCs ranging from 0.72 to 0.86 and 0.64 to 0.78, respectively.

**Table 1:** Diagnostic accuracy of Circulating MiRNAs for MASLD

MASLD	AUROC	References
miR-122	0.67 / 0.65 / 0.77 / 0.85 / 0.72 / 0.79/ 0.82 / 0.85	[19, 25, 34, 55, 59, 69, 85, 92]
miR-128	0.76 / 0.71	[19, 96]
miR-200	0.96	[19]
miR-298	0.98	[19]
miR-342	0.94	[19]
miR-34a	0.79 / 0.72 /0.78 / 0.86	[22, 24, 55, 92]
miR-192	0.64 / 0.78 /0.65 / 0.71	[22, 49, 59, 96]
miR-6888-5p	0.91	[23]
miR-4488	0.79	[28]
miR-379	0.72	[32]
miR-1290	0.62	[59]
miR-125b	0.66	[55]
miR-27b-3p	0.69	[59]
miR-99a-5p	0.54 / 0.60	[59, 68]
miR-148a-3p	0.55	[59]
miR-146a	0.80	[68]
miR-640	0.61	[68]
miR-135a	0.84	[69]
miR-129b-5p	0.69	[69]
miR-504-3p	0.70	[69]
miR-let-7d-5p	0.85	[83]
miR-375	0.88	[92]
miR-16	0.86	[92]
miR-21	0.83 / 0.69	[92] / [55]
miR-193a-5p	0.92	[95]

Abbreviations: MASLD, Metabolic Dysfunction-Associated Steatotic Liver Disease; AUROC, Area Under the Receiver Operating Characteristic curve

In the case of MASH (Table 2), several miRNAs demonstrated high diagnostic accuracy, with miR-200, miR-298, and miR-342 achieving AUROCs of 0.99, indicating near-perfect diagnostic potential. MiR-122 also showed significant accuracy, with AUROCs of 1 and 0.81 in different studies. MiR-34a demonstrated moderate to high accuracy with AUROCs ranging from 0.81 to 0.84. Other miRNAs, such as miR-181d and miR-99a, achieved AUROCs of 0.86 and up to 0.91, respectively, highlighting their diagnostic relevance. It is important to emphasize the recurrence of certain miRNAs across multiple studies, such as miR-122, miR-34a, miR-128 and miR-192, as their repeated association may indicate a key role in the pathophysiological mechanisms analyzed.

**Table 2:** Diagnostic accuracy of circulating MiRNAs for MASH

MASH	AUROC	References
miR-122	1 / 0.81	[19, 108]
miR-128	0.90	[19]
miR-200	0.99	[19]
miR-298	0.99	[19]
miR-342	0.99	[19]
miR-34a	0.81 /0.84	[36, 108]
miR-16	0.71	[36]
miR-197	0.77	[58]
miR-146b	0.75	[58]
miR-181d	0.86	[58]
miR-99a	0.76 /0.91	[58, 108]
miR-21-5p	0.73	[30]
miR-151a-3p	0.75	[30]
miR-192-5p	0.77	[30]
miR-4449	0.74	[30]

Abbreviations: MASH, Metabolic Dysfunction-Associated Steatohepatitis; AUROC, Area Under the Receiver Operating Characteristic curve

For HCC (Table 3), miR-214 stood out with a high AUROC of 0.88, while miR-34a demonstrated consistent performance with AUROCs ranging from 0.73 to 0.76. Other individual miRNAs, such as miR-19-3p and miR-16-5p, showed moderate accuracy with AUROCs of 0.82 and 0.74, respectively.

**Table 3:** Diagnostic accuracy of circulating MiRNAs for HCC

HCC	AUROC	References
miR-19-3p	0.82	[26]
miR-16-5p	0.74	[26]
miR-223-3p	0.65	[26]
miR-30d-5p	0.72	[26]
miR-451a	0.70 /0.60	[26, 79]
miR-214	0.88	[40]
miR-142-5p	0.62	[79]
Let-7f-5p	0.64	[79]
miR-122-5p	0.59	[79]
miR-29a-3p	0.59	[79]
miR-34a	0.73–0.76	[93]
miR-122	0.76	[85]

Abbreviations: HCC, Hepatocellular Carcinoma; AUROC, Area Under the Receiver Operating Characteristic curve

The diagnostic accuracy of circulating miRNA panels for various stages of MASLD, including MASLD, MASH, and HCC, has been extensively evaluated in recent studies. These miRNA panels have shown considerable potential in improving the diagnostic precision for MASLD subtypes.

Several miRNA panels have demonstrated varying degrees of diagnostic performance (Table 4). A panel consisting of miR-18a/miR-16, miR-25-3p/miR-16, miR-18a/miR-21-5p, and miR-18a/miR-92a-3p yielded an AUROC of 0.88, suggesting high diagnostic accuracy for MASLD detection. Another panel comprising miR-1290, miR-27b-3p, miR-192-5p, miR-99a-5p, miR-148a-3p, and miR-122-5p also exhibited strong diagnostic potential, with an AUROC of 0.85. Other panels, which obtained an AUROC of 0.85 after 12 and 24 months of nutritional intervention, were composed of miR-29b-3p, miR-122-5p, miR-151a-3p, and BMI, and by miR-21-5p, miR-151a-3p, and BMI, respectively. Additional panels, including miR-33b and miR-1220, demonstrated an AUROC of 0.85, while a combination of waist circumference (WC), ALT, glycemia, and miR-331 resulted in an AUROC of 0.80. The best AUROC values obtained for MASLD prediction using miRNA panels were from those composed of changes in liver stiffness, in chemerin, in depressive symptoms, in miR-122-5p, and in BMI (AUROC = 0.86) and from the panel including changes in hepatic fat content (%), changes in total cholesterol (TC), in triglycerides (TG), in miR-15b-3p, and in BMI (AUROC 0.89).

**Table 4:** Diagnostic accuracy of MiRNA panels for MASLD patients

miRNA panels		
MASLD	AUROC	References
miR-18a/miR-16; miR-25-3p/miR-16; miR-18a/miR-21-5p; miR-18a/miR-92a-3p	0.88	[41]
miR-1290, miR-27b-3p, miR-192-5p, miR-99a-5p, miR-148a-3p, Mir-122-5P	0.85	[59]
miR33b, miR1220	0.85	[75]
WC, ALT, glycemia, miR-331	0.80	[78]
miR-126-5p, miR-15b-3p and BMI	0.68	[89]
miR29b-3p, miR-122-5p, miR151a-3p and BMI	0.85	[89]
miR21-5p, miR151a-3p and BMI	0.85	[89]
∆ Liver stiffness, ∆ Chemerin, ∆ depressive symptoms, ∆ miR-122-5p and ∆ BMI	0.86	[90]
∆ Hepatic fat content (%), ∆ TC, ∆ TG, ∆ miR-15b-3p and ∆ BMI	0.89	[90]
**MASH**	AUROC	References
miR-21-5p, miR-151a-3p, miR-192-5p, and miR-4449	0.87	[30]
MiR-122, miR-192, miR-21	0.81	[81]
miR-122, miR-192, miR-21, CK18-Asp396	0.83	[81]
HCC	AUROC	References
miR-34a, miR-221 miR-16, miR-23-3p, miR-122-5p, miR-198, miR-199a-3p (diabetic liver cirrhosis)	0.99	[20]
miR-34a, miR-221 miR-16, miR-23-3p, miR-122-5p, miR-198, miR-199a-3p (diabetic nonmalignant)	0.96	[20]
miR-181a + FIB-4	0.75	[77]
Let-7f-5p, miRNA-122-5p, miRNA-142-5p, miRNA-29a-3p, miRNA-451a	0.75	[79]
miR-122, miR-192, miR-375	0.70	[106]

Abbreviations: MASLD, Metabolic Dysfunction-Associated Steatotic Liver Disease; MASH, Metabolic Dysfunction-Associated Steatohepatitis; HCC, Hepatocellular Carcinoma; AUROC, Area Under the Receiver Operating Characteristic curve; WC, Waist Circumference; ALT, Alanine Aminotransferase; miRNA, microRNA; TC, Total Cholesterol; TG, Triglycerides

For MASH, miRNA panels have similarly shown promising results. A panel consisting of miR-21-5p, miR-151a-3p, miR-192-5p, and miR-4449 achieved an AUROC of 0.87, demonstrating its diagnostic utility for MASH. Another panel, which included miR-122, miR-192, and miR-21, displayed an AUROC of 0.81, and the addition of CK18-Asp396 increased the AUROC to 0.83, further underscoring the utility of combining miRNAs with other biomarkers to enhance diagnostic accuracy in MASH. For HCC, miRNA panels have exhibited exceptional diagnostic accuracy in distinguishing HCC from other liver conditions. A panel comprising miR-34a, miR-221, miR-16, miR-23-3p, miR-122-5p, miR-198, and miR-199a-3p, evaluated in diabetic liver cirrhosis patients, achieved an outstanding AUROC of 0.99. In diabetic non-malignant patients, the same panel demonstrated an AUROC of 0.96. Additionally, a panel containing let-7f-5p, miR-122-5p, miR-142-5p, miR-29a-3p, and miR-451a achieved an AUROC of 0.75, indicating moderate diagnostic accuracy. Furthermore, a combination of miR-122, miR-192, and miR-375 exhibited an AUROC of 0.70, suggesting potential but less robust diagnostic capability in the context of HCC.

### MiRNAs associated with the severity of the disease and its progression

Several circulating miRNAs have been associated with the prognosis of MASLD, showing potential to predict disease severity and progression over time.

Serum miR-122 dynamics have been highlighted as a potential prognostic marker in MASLD, particularly in patients with advanced stages of fibrosis who do not show improvements in stage scores [29]. Elevated levels of serum miR-122, in conjunction with the Fibrosis-4 (FIB-4) index, are significant risk factors for mortality in MASLD patients, as demonstrated in Japanese cohorts [33]. Early detection of these biomarkers could lead to improved prognosis through early intervention. MiR-122 is also linked with hepatic steatosis and fibrosis [88]. Patients with mild steatosis (< 33%) exhibit significantly lower levels of hepatic miR-122 compared to those with severe steatosis (> 33%) [39].

Similarly, serum miR-21 levels were significantly higher in MASH patients as well as hepatic miR-223 expression was elevated in severely obese MASH patients compared to both MASL and healthy controls [81, 97]. Serum levels of miR-21 were significantly reduced in patients with MASLD when compared to healthy controls [56]. In MASH liver biopsies, miR-141 and miR-200c were found to be significantly upregulated. This upregulation was observed in both human patients and animal models, emphasizing the clinical relevance of miR-141 and miR-200c as potential biomarkers for MASH diagnosis and progression [101].

The levels of hepatic miR-9 were significantly elevated in the MASH group compared to non-MASH, further highlighting its potential as a biological marker for disease severity and progression [54], as well as the expression of serum miR-182, miR-301a, and miR-373 has been found elevated in both serum and ascitic fluid of MASH patients [31].

In contrast, serum miR-103 levels were notably elevated in the MASLD cohort, exhibiting a positive correlation with HOMA-IR, TG, and body mass index (BMI), all of which were identified as independent risk factors for MASLD [57]. In addition, hepatic miR-103a-2, miR-106b, miR-576-5p, miRPlus-1137, miR-892a, miR-1282, miR-3663-5p, and miR-3924 are all upregulated in MASLD, with potential for use as biomarkers in assessing disease progression and prognosis [80]. The specific deregulation of miRNAs, such as the increase of miR-33a and miR-224, is associated with hepatic steatosis, while the expression of miR-221 decreases independently of etiology, reflecting molecular changes linked to disease progression [91].

Hepatic overexpression of miR-34a resulted in impaired insulin signaling and disrupted glucose metabolism in hepatocytes, further implicating miRNAs in the metabolic dysregulation associated with MASLD [61].

Hepatic miR-363-5p expression levels have been linked to the prognosis of HCC. The Kaplan-Meier test revealed that low expression of miR-363-5p was associated with better overall survival in HCC patients, suggesting its potential as a prognostic biomarker for patient outcomes in liver cancer [35]. Hepatic miR-483-5p has shown significant downregulation in HCC tumors compared to normal liver tissues [100]. The reduction in miR-26a expression is common in hepatocellular carcinomas and is associated with increased tumor recurrence and mortality, reflecting its involvement in the progression of liver disease [68].

In the context of coronary artery disease (CAD), patients with MASLD and concurrent CAD demonstrated lower plasma levels of miR-132 and higher levels of miR-143 compared to MASLD patients without CAD, indicating a potential miRNA dysregulation in the cardiovascular comorbidities associated with MASLD [98]. Additionally, obese MASLD patients exhibited decreased circulating plasma levels of miR-145, miR-211, miR-146a, and miR-30c when compared to their lean counterparts, while higher levels of miR-161 and miR-241 were detected in obese individuals, further highlighting the influence of adiposity on miRNA profiles in MASLD [98].

These miRNAs among others, represent promising biomarkers for monitoring disease progression in MASLD, from its early stages to more severe forms like MASH and HCC. Their potential for predicting long-term outcomes and guiding treatment decisions in MASLD patients is evident, though further validation in larger cohorts is necessary.

### Changes in MiRNA levels in different MASLD interventions or treatments

In exploring the role of miRNAs in MASLD and related conditions, various therapeutic interventions, including surgery, supplementation, and dietary interventions, have shown the ability to alter miRNA profiles in ways that may either mitigate or exacerbate the condition.

Laparoscopic bariatric surgery is the most effective approach for weight loss in morbid obesity and has also been shown to improve liver function in biopsy-confirmed MASLD/MASH. In patients with MASLD undergoing liver surgery, a significant upregulation of miR-200c-3p has been observed, indicating a potential protective role through the modulation of dual-specificity protein phosphatase 1 (DUSP1) expression and MAPK activity. Following the laparoscopic sleeve gastrectomy (LSG) procedure, miR-200c-3p expression significantly decreased, corresponding with the improvement of MASLD, suggesting a beneficial effect of the surgery in modulating miRNA levels and promoting disease remission [43].

Vitamin D supplementation could have a beneficial effect on MASLD by reducing liver fibrosis markers, fibrogenic miRNAs, and improving liver function and metabolic factors. A 12-week supplementation regimen led to a significant reduction in the expression of miR-21 and miR-122, two miRNAs implicated in the progression of liver disease, in comparison to a placebo group. This supports the potential of vitamin D as a modulator of miRNA expression [60].

Vitamin E has preventive and protective effects on MASLD by improving hepatic steatosis (FLI, L/S ratio), insulin resistance (HOMA-IR), oxidative stress (MDA), inflammation (hs-CRP, TNF-α, IL-6, leptin, adiponectin), and hepatocyte apoptosis (CK18-M30). The effects of different forms of vitamin E, specifically δT3 and αTF, have been studied for their ability to regulate miRNA expression. Both forms significantly downregulated miRNAs such as miR-122, miR-21, miR-103a-2, miR-421, miR-375, and miR-34a, which are involved in inflammation and apoptosis. δT3, in particular, showed greater efficacy than αTF in reducing the expression of miR-375 and miR-34a, further highlighting the potential of vitamin E derivatives in modifying miRNA profiles and, by extension, influencing disease mechanisms in MASLD [62].

Mastiha, an herbal supplement, has anti-inflammatory and lipid-lowering properties, suggesting a hepatoprotective effect in MASLD patients. It reduces serum lipid and liver enzyme levels, inhibiting inflammatory cytokines and pathways related to inflammation and oxidation, such as the NF κB pathway. Its main components, particularly triterpenes, are responsible for these beneficial effects. The role of Mastiha in modulating miRNA expression has also been explored. In patients with less advanced fibrosis, Mastiha prevented the increase in miR-155, a miRNA that plays a crucial role in T helper-17 (Th17) differentiation and function [86]. Mastiha intervention, by influencing miRNAs regulating inflammation, lipid metabolism, and oxidative stress, could offer a promising therapeutic approach to improving MASLD progression and preventing further complications.

Omega-3 polyunsaturated fatty acids (n-3 PUFAs) have shown beneficial effects in MASLD by improving metabolic, inflammatory, and histological profiles. Docosahexaenoic acid **(**DHA) is effective in inhibiting lipogenic enzymes, increasing fatty acid oxidation, and reducing pro-inflammatory cytokines, contributing to a reduction in liver fat accumulation and inflammation [109, 110] (94,94). Dietary interventions, particularly supplementation with n-3 PUFAs, demonstrated that after six months of fish oil supplementation, n-3 PUFAs were incorporated into erythrocytes, leading to a reduction in alkaline phosphatase (ALP) levels and liver fibrosis without significantly altering circulating miR-122 expression in MASLD individuals. This suggests that while n-3 PUFAs may not directly impact miR-122, they could still exert beneficial effects on liver health through other mechanisms [107]. Several studies have suggested that the beneficial effects of n-3 PUFAs on MASLD could be mediated by the n-3 PUFAs-derived specialized proresolving lipid mediators, such as resolvins, protectins and maresins. Indeed, low serum levels of Maresin 1 (MaR1) have been associated with MASLD in humans [111]. Moreover, several preclinical trials have evidenced that MaR1 treatment attenuates MASLD in mice models [112, 113]. Interestingly, it has been shown that some of these beneficial actions of MaR1 on hepatocytes were associated with specific miRNA signatures, targeting both protein folding and apoptosis [114]. Recent evidence suggests that certain microRNAs, such as miR-21 and miR-146b, are upregulated during the resolution phase of inflammation, while others like miR-142-5p are downregulated. These temporal patterns highlight the role of miRNAs in inflammation resolution. Although direct data on MaR1 and its modulation of these specific miRNAs in MASLD is limited, it is plausible that MaR1 may influence similar pathways, given its pro-resolving properties [115].

The dietary intervention, characterized by high adherence to the Mediterranean dietary pattern, was able to modulate the expression of circulating miRNAs after 6, 12, and 24 months. Logistic regression analyses showed that the most effective panels for diagnosing MASLD resolution after the nutritional intervention included miR-15b-3p, miR-126-5p, and BMI at 6 months; miR-29b-3p, miR-122-5p, miR-151a-3p, and BMI at 12 months; and miR-21-5p, miR-151a-3p, and BMI at 24 months. These panels demonstrated good diagnostic performance, highlighting the potential of circulating miRNAs combined with clinical variables in monitoring MASLD improvement following a Mediterranean diet [89]. In addition, this diet was able to identify other panels integrating miR-15b-3p with metabolic and clinical parameters such as HDL-c, body mass index, triglycerides, and depressive symptoms have demonstrated high accuracy in predicting the presence and severity of MASLD over 24 months, highlighting the importance of multivariable combinations for a more comprehensive clinical assessment [90].

### Functional pathways and biological processes in which MiRNAs are implicated

This review found 24 studies that investigated key gene pathways and biological processes implicated in metabolic dysfunction and liver disease.

The LPS (Lipopolysaccharide)/TLR-4 (Toll-like receptor 4)/FoxO3 (Forkhead Box O3) pathway was frequently highlighted, alongside TMBIM1, AMPK and PPARα signaling. Key transcription factors such as STAT3, TCF3, RELA, RUNX1, SMAD2/3-SMAD4, and NF-κB (including NFKB1 and RELB) were identified in TF-miR networks, suggesting miRNA involvement in gene regulation.

MiR-582-3p has been shown to promote MASH progression by regulating the gut microbiota and modulating *TMBIM1* [27]. MiR-21-5p/PPARα pathway was found to be deregulated in patients with MASH-related HCC, correlating with worse prognosis. This pathway’s disruption is associated with the progression from MASLD to MASH, ultimately leading to liver cancer [76]. In MASLD patients, increased levels of miR-223 in the liver were observed, along with a decrease in *FOXO1* mRNA suggesting a potential role for miR-223 in the regulation of metabolic processes in the liver [63]. Dysregulation of miR-135a-3p is linked to the AMPK signaling pathway, a key regulator of energy metabolism, and shows promise as a non-invasive biomarker for MASLD diagnosis [69].

Additionally, the TNF and p53 signaling pathways were common, with regulatory involvement from genes such as *RHGAP1*, *SLC10A1*, and *SIX5*. P53 appears to contribute to liver steatosis in MASH patients by inducing the miR-34a-HNF4α pathway. In MASH patients, miR-34a was induced significantly, while other miRNAs, such as miR-19b and miR-27b, were not. Moreover, p53 levels were notably increased in MASH livers, emphasizing its role in miRNA regulation and liver disease progression [103]. On the other hand, miR-378 is transcribed independently of *Ppargc1β* and is selectively activated by LXRα, which also inhibits Ppargc1β. This dual effect impairs fatty acid oxidation and promotes lipogenesis, contributing to LXRα-induced hepatosteatosis—a key factor in MASH development [73].

Inhibition of miR-423-5p, let-7a-5p, and let-7c-5p can restore the classic IL-6 signaling pathway in the liver, highlighting their role in modulating inflammation. Interleukin-6 (IL-6) is a multifunctional cytokine with a crucial role in inflammation, influencing the pathogenesis of various diseases, including MASLD. Elevated levels of IL-6 in serum, liver, and adipose tissue in patients with MASH have been linked to insulin resistance, steatosis, and liver injury. Lastly, myeloid-specific IL-6 signaling has been implicated in liver fibrosis regulation. It has been shown that this signaling pathway can inhibit fibrosis through the exosomal transfer of anti-fibrotic miR-223 into hepatocytes, offering a novel therapeutic target for MASLD. By modulating miR-223 levels, IL-6 signaling may provide a mechanism for controlling fibrosis progression in MASLD [47]. Serum exosomal miRNAs such as miR-let-7b-5p, miR-378 h, miR-1184, miR-3613-3p, miR-877-5p, miR-602, miR-133b and miR-509-3p, demonstrate inverse expression patterns with their target mRNAs, reflecting regulatory interactions crucial in MASLD progression. Notably, in fibrosis, multiple miRNA–mRNA pairs showed anticorrelated expression, highlighting their potential as non-invasive biomarkers for disease severity and as therapeutic targets [67].

Furthermore, miR-423-5p and other miRNAs, such as miR-155-5p, miR-200c-3p and miR-21 have been shown to downregulate the expression of genes like *TGFB1* and *SMAD7*, which are involved in the TGF-β signaling pathway, suggesting their role in fibrosis regulation [84, 105]. Additionally, miR-423-5p regulates hepatic metabolism by repressing *FAM3A* [53], while miR-9, whose levels increase with the severity of MASLD, may affect several key genes, such as *SIRT1* and *CoREST*, promoting disease progression, which were recognized for their roles in liver metabolism, inflammation, and stress response [54].

Liver-specific metabolic pathways also featured prominently, with SFRP5, PPARγ, and CD36 identified as critical modulators. MiR-20a-5p protects against metabolic disorders by targeting CD36, suggesting its potential as a biomarker for MASLD [46]. MiR-21-5p is associated with the low expression of PPARγ, an important gene in lipid metabolism, and is proposed as a therapeutic target through the regulation of SFRP5, an anti-inflammatory adipokine [45].

MiR-223 is crucial in controlling the progression from steatosis to MASH by inhibiting *Cxcl10* and *Taz* expression in the liver [97]. Additionally, miR-132 plays a key role in hepatic lipid homeostasis by suppressing multiple targets, and its reduction may offer therapeutic potential for hepatic steatosis [102].

Other notable pathways included insulin-like growth factor 1 (IGF-1), AKT serine/threonine kinase (AKT), WNT pathway (with impacts on β-catenin and p-53 expression), NF-κB activation, and autophagy pathways. NF-κB activation, autophagy, and negative regulation of the apoptotic process are the main potential underlying mechanisms regulated by miR-146b, miR-194, miR-214 in liver fibrosis pathogenesis [40].

MiR-34a and miR-29a are involved in the WNT/β-catenin signaling pathway, with dysregulated expression in MASH patients. In these individuals, miR-34a and circRNA-HIPK3 are overexpressed, while miR-29a and circRNA-0046367 are significantly reduced compared to healthy controls. This imbalance affects lipid metabolism, contributing to disease progression. Additionally, the circRNA_0046367/miR-34a/PPARα regulatory system is disrupted in hepatic steatosis, with elevated miR-34a inhibiting PPARα, a key lipid metabolism regulator [64].

MiR-192, which is significantly downregulated in MASLD, could positively regulate the gene *MT1X*. MiR-30e negatively regulates the gene *VIM*, which plays a key role in lymphocyte adhesion and transmigration. MiR-15a, also downregulated, could positively regulate the gene *PDIA6*, involved in binding unfolded proteins in the endoplasmic reticulum. Additionally, the expression of the *P8* module is associated with miR-122 and miR-24-2 [38].

MiR-181a is elevated in MASLD and directly acts on PPARα, regulating lipid metabolism, which suggests that inhibiting miR-181a could be a therapeutic strategy [51]. MiR-181b was also upregulated in MASLD, leading to the downregulation of *SIRT1*, a gene that promotes hepatic lipid metabolism. In vitro and in vivo studies revealed that inhibiting miR-181b expression alleviated hepatic steatosis, while overexpressing *SIRT1* blocked the pathological effects of miR-181b, suggesting that targeting miR-181b could be a therapeutic strategy for MASLD [65].

Moreover, miR-30a-3p was found to be significantly elevated in MASLD patients, and it regulated the degree of steatosis by directly targeting *PPAR*-α, further linking miRNAs to lipid metabolism in the liver [66].

In MASLD patients, lncRNA plasmacytoma variant translocation 1 (PVT1) is upregulated, while miR-20a-5p is downregulated. PVT1 may regulate the expression of miR-20a-5p by binding to its 3’-UTR, which in turn affects insulin sensitivity and lipid metabolism, contributing to the development of MASLD [44].

MiR-30a-3p carried by sEVs from steatotic hepatocytes drives foam cell formation and atherogenesis through ABCA1 regulation. Targeting miR-30a-3p or its vesicle-mediated delivery could be a therapeutic strategy to slow MASLD-associated atherosclerosis [72].

## Discussion

This review highlights the essential role of miRNAs in MASLD, emphasizing their potential as valuable biomarkers for the diagnosis, prognosis, and treatment of the disease. miRNAs offer several advantages over traditional biomarkers, as they are stable in serum and plasma, facilitating their use in minimally invasive, accessible, and cost-effective tests for early diagnosis and monitoring of MASLD, thus eliminating the need for repeated biopsies. This stability makes them an ideal option for clinical implementation.

In terms of diagnosis, several miRNAs have shown the ability to distinguish between different stages of the disease, potentially enabling earlier diagnosis, particularly in high-risk populations. However, the variability in results depending on the population and disease stage reinforces the need for validation in larger and more diverse cohorts. The diagnostic utility of miRNAs in MASLD exhibits considerable heterogeneity, influenced by factors such as the type of biological sample, the characteristics of the studied cohorts such as age, sex or metabolic comorbidities, and the methodologies employed. These variations partly explain the wide range of AUROC values reported in the literature, such as in the case of miR-122, which ranges from 0.65 to 1.0.

The diagnostic accuracy of circulating miRNAs for MASLD shows promising results across various disease stages. For early MASLD, miR-200 and miR-298 demonstrated outstanding performance with AUROCs of 0.96 and 0.98, respectively, while miR-342, miR-6888-5p, miR-193a-5p, miR-135a or miR-122 achieved AUROCs above 0.84–0.90. The expression of miR-193b is directly linked to the secretion of adiponectin, a protein secreted by adipocytes, which enhances hepatic insulin sensitivity [116]. Previous research has shown that miR-122-5p levels are elevated and positively associated with markers of MASLD severity, as well as with body weight, triglycerides, and insulin resistance. Additionally, it has been found to differentiate MASLD patients from healthy individuals [25, 117].

In MASH, miR-200, miR-298, and miR-342 reached near-perfect AUROCs of 0.99, and miR-181d and miR-99a exhibited strong accuracy with AUROCs of 0.86 and 0.91. For HCC, miR-214 stood out with an AUROC of 0.88, while miR-19-3p and miR-16-5p showed moderate accuracy. miR-181d may contribute to regulating lipid content in hepatocytes. It has been shown to reduce the number of lipid droplets and decrease cellular TG and cholesterol esters in cell cultures [118]. miRNA-99a is a much stronger predictor for MASH development [108]. A study reported that the AUC for the miRNA panel of miRNA-99a and miRNA-122 reached 0.89, highlighting its potential clinical significance [108]. Numerous miRNAs such as miR-15b, miR-21, miR-23a, miR-26a, miR-155, miR-200c, miR-222, miR-224, miR-374a, miR-423, let-7a, and let-7c have great potential as diagnostic biomarkers of MASH in serum [84].

Panels of miRNAs also demonstrated robust diagnostic potential. A panel including miR-18a/miR-16 and miR-18a/miR-92a-3p yielded an AUROC of 0.88 for MASLD detection [22]. Another combination of miRNAs, such as miR-1290, miR-27b-3p, and miR-122-5p, achieved an AUROC of 0.85 [41]. These findings highlight the potential of miRNAs, both individually and in panels, to enhance diagnostic precision for MASLD and its advanced stages. Panels including miR-15b-3p, miR-126-5p, and BMI reached an AUC of 0.68 at 6 months; panels with miR-29b-3p, miR-122-5p, miR-151a-3p, and BMI showed an AUC of 0.85 at 12 months; and panels including miR-21-5p, miR-151a-3p, and BMI achieved an AUC of 0.85 at 24 months [89]. Additionally, broader panels incorporating liver stiffness, HDL-c, BMI, depressive symptoms, and triglycerides yielded an AUC of 0.90, while another panel including hepatic fat content, total cholesterol, miR-15b-3p, triglycerides, and depressive symptoms reached an AUC of 0.89 at 24 months [90]. The miRFIB-score, a panel which consist of miRNA-142-5p, miRNA-451a, Let-7f-5p, miRNA-122-5p, and miRNA-29a-3p, had a predictive value superior to the clinical scoring systems FIB-4, APRI, and AST/ALT, although accessible and validated, they lack accuracy for identifying early stages and minor changes in liver fibrosis [79]. MiRNA-1290 is also linked to various cancers, including laryngeal squamous cell carcinoma, cervical cancer, and pancreatic cancer [119]. In this regard, miRNAs could offer an additional advantage as sensitive and specific markers, especially if methods are standardized and validated in multicenter cohorts.

The use of miRNA panels in diagnosing MASLD subtypes (MASLD, MASH, and HCC) improves diagnostic accuracy compared to individual miRNAs. These panels hold significant potential as minimally invasive biomarkers, contributing to better clinical management and early detection of MASLD. However, further validation and standardization across diverse populations are needed for their implementation in clinical settings. By integrating multiple miRNAs, these panels enhance diagnostic performance compared to individual markers, offering a minimally invasive and potentially cost-effective alternative for early detection and management of MASLD. However, further validation studies across diverse cohorts and clinical settings are warranted to standardize these panels and facilitate their translation into routine clinical practice.

From a prognostic perspective, miRNAs could be key in identifying patients at higher risk of progressing to severe forms of the disease, such as cirrhosis or HCC. While further research is required to confirm their long-term predictive capabilities, miRNAs have the potential to improve prognostic models and guide personalized therapeutic decisions.

Several studies suggest that miR-122, miR-33, miR-34a, and miR-21 play key roles in regulating liver metabolism [120, 121]. MiRNA-122 is one of the most abundant miRNAs in the liver and is essential for regulating lipid metabolism. Furthermore, the levels of miRNA-122 positively correlate with the severity of MASLD [15, 71]. Inhibiting miR-122 leads to a decrease in the mRNA expression of several lipogenic genes and an improvement in liver steatosis [122]. In addition, serum levels of miR-122 also tended to decrease before the progression of fibrosis [37].

Partial least squares (PLS) models combining serum miR-145-3p, miR-122-5p, miR-143-3p, miR-500a-5p, and miR-182-5p effectively predicted hepatic steatosis, showing low error rates in cross-validation and external validation. This supports the potential of these miRNAs as non-invasive prognostic biomarkers for MASLD [88].

MiR-21-5p increases in the plasma of patients with MASH and it is associated with hepatic metabolism, inflammation, and lipid metabolism [81, 123]. MiR-34a level was positively correlated with histopathological features of the liver [108].

Other miRNAs play pivotal roles in the progression, regulation, and associated conditions of MASLD and MASH. MiR-9 is predominantly expressed in the brain, liver, and pancreatic islets, where it facilitates insulin exocytosis [124]. MiR-150-5p has been linked to hepatic fibrosis [125], while miR-141/200c deficiency correlates with reduced phosphatidylcholine (PC) levels, aligning with MASLD’s known association with decreased PC in humans [126]. MiR-192-5p, highly expressed in the kidneys, intestine, and adipose tissue, regulates lipid synthesis in MASLD, is present in hepatocyte-derived exosomes, and contributes to MASLD progression. Additionally, miR-192 functions as an oncogene in cancers like colon, breast, and gastric cancers [127, 128, 129, 130].

MiR-128 regulates apoptosis, fatty acid synthesis, and cholesterol metabolism pathways, and acts as a tumor suppressor in cancers such as HCC. Dysregulation of miR-128-3p has also been reported in these contexts [131–133]. Reduced miR-26a expression is frequently observed in HCC patients with various liver diseases and is associated with higher recurrence and mortality post-surgery [104]. MiRNAs such as miR-2861, miR-3940-5p, miR-6727-5p, miR-6771-5p, miR-6780b-5p, miR-6845-5p, and miR-7114-3p have been associated with MASH and MASLD, emphasizing their relevance in disease progression [134]. Serum miR-486-5p levels show a strong positive correlation with fatty liver detected via ultrasound [135], and miR-181b expression increases in advanced MASLD [65].

MiR-27a and miR-27b exhibit contrasting roles in MASLD. While miR-27b is elevated in MASLD, miR-27a reduces lipid accumulation in hepatocytes by inhibiting fatty acid synthesis, enhances TG hydrolysis, and facilitates free fatty acid release [136, 137]. MiR-483, crucial in MASLD and HCC regulation, is downregulated in HCC across various populations compared to healthy individuals [100]. These findings underscore the intricate roles of miRNAs in lipid metabolism, liver pathology, and their potential as biomarkers for MASLD, MASH, and HCC.

Regarding treatment, miRNAs could not only be used to monitor treatment response but also to develop targeted therapies that modulate the expression of specific miRNAs involved in disease progression. The ability of miRNAs to influence various biological processes, such as inflammation, fibrosis, and lipid metabolism, opens new possibilities for more precise and effective therapeutic approaches. However, their clinical application in treatment remains an area under development that requires further studies and clinical trials to determine their efficacy. Various treatments have shown the ability to modify miRNA profiles in MASLD, suggesting their therapeutic potential. Interventions such as bariatric surgery, supplementation with vitamins (D and E), herbal supplements like Mastiha, Mediterranean diet and n-3 PUFA administration can influence the expression of key miRNAs involved in lipid metabolism regulation, inflammation, and hepatic fibrosis [62, 86, 89, 90, 108]. These treatments not only act as potential modulators of miRNAs but also open new avenues for therapeutic management of the disease, highlighting the role of miRNAs as targets in MASLD treatment.

Additionally, a study showed that miRNAs such as miR-4449, which is upregulated in obesity and decreases after bariatric surgery, miR-199a-3p, which regulates liver fat production, and miR-200b, upregulated in diet-induced MASLD, have been associated with potential therapeutic effects. The expression of miR-4449 is not well-known in patients with MASLD. In individuals with obesity, circulating exosomal miR-4449 showed increased expression compared to the healthy control group, and its expression decreased after bariatric surgery [138], while miR-199a-3p regulates liver fat production by targeting *SP1*, suggesting a treatment for MASLD [139]. On the other hand, miR-200b, known for its tumor suppressor activity, was found to be upregulated in diet-induced murine models of MASLD [140, 141].

MiR-29a has also been shown to reduce obesity and alleviate steatosis and fibrosis, further suggesting its relevance in MASLD treatment [142]. Serum miR-29b was associated with intrahepatic lipid content and MASLD in a Chinese population-based study [70]. Moreover, miR-223 plays a role in minimizing liver inflammation by targeting neutrophils and may offer a mechanism for controlling liver damage [143–145] and miR-223 impairs the compensatory response of β-cells to high-fat-diet-induced insulin resistance by directly targeting *FOXO1* [146]. Furthermore, miRNAs such as miR-29b-3p, miR-122-5p, miR-151a-3p, and miR-21-5p have shown promising results in predicting MASLD progression, demonstrating their diagnostic and therapeutic potential [89].

Therapeutic interventions, ranging from surgical procedures to supplementation and dietary changes, have the potential to influence miRNA expression in MASLD. These findings underscore the importance of miRNAs as both biomarkers and therapeutic targets in the treatment and management of liver diseases like MASLD, providing avenues for future research and clinical application.

On the other hand, numerous signaling pathways play pivotal roles in the pathogenesis of MASLD, influencing key processes such as lipid metabolism, inflammation, and fibrosis. Understanding these pathways is crucial not only for elucidating the molecular mechanisms underlying disease progression but also for identifying novel biomarkers and therapeutic targets. The LPS/TLR-4/FoxO3 pathway, PPARα signaling, and TNF and p53 pathways were frequently identified, with miRNAs such as miR-21-5p, miR-223, and miR-34a playing central roles. MiR-21-5p is involved in lipid metabolism through the PPARα pathway, while miR-223 regulates liver inflammation and fibrosis. The regulatory protein SMAD7, crucial in TGF-β signaling, is a target of miR-21 and has been shown to counteract its regulatory effects [147, 148].

In MASH livers, inflammatory environments are thought to enhance TGF-β signaling, which subsequently stimulates *SMAD2/3* phosphorylation and their interaction with *SMAD4* [105, 147]. miR-21-5p is induced in MASH progression and recognized as a biomarker in patients with MASH [149]. The absence of miR-21-5p results in a significant reduction in fibrogenesis, TGF-β production, and the downstream signaling of the TGF-β pathway [105].

In addition, miR-34a is associated with liver steatosis by targeting the p53 signaling pathway. Serum miR-34a strongly correlates with liver inflammation severity and promotes cholesterol accumulation by downregulating *SIRT1* and dephosphorylating *AMPK* [150, 151]. CircRNA_0046367 likely alleviates hepatocellular steatosis by enhancing miR-34a/PPARα signaling, which improves *PPARα*-mediated lipid metabolism [64]. P53, an oxidative stress-inducible protein, has been shown to upregulate miR-34a expression [103]. miR-20a-5p may enhance the synthesis of liver glycogen and play a role in hepatic insulin signaling triggered by elevated glucose levels. It achieves this by targeting *p63*, which in turn regulates the expression of *p53* and *PTEN* [152]. Circulating miR-20a may serve as a predictive marker for liver fibrosis associated with hepatitis C virus (HCV) [153].

Other key miRNAs, such as miR-423-5p and miR-9, modulate inflammation and fibrosis through the TGF-β and IL-6 pathways. Metabolic processes like insulin sensitivity, lipid metabolism, and hepatic lipid homeostasis are influenced by miRNAs like miR-20a-5p, miR-132, and miR-181b, which regulate genes such as *CD36*, *SFRP5*, and *PPARγ*. Gupta et al. showed increased expression of miR-181a in the tissue of cirrhotic livers, possibly associated with increased fibrogenesis via TGF-β [154].

Additionally, miR-29a and miR-192 are involved in the WNT/β-catenin signaling pathway, affecting lipid metabolism and contributing to disease progression. Other miRNAs such as miR-122-5p, miR-335-5p, and miR-27a are related to the insulin signaling pathway and WNT signaling pathway [155]. The WNT family is highly expressed in the liver, and studies show that it signaling pathway activates hepatic stellate cells (HSCs), contributing to nonalcoholic fatty liver disease and liver fibrosis [156]. The molecules targeted by miR-335-5p are involved in controlling lipid and carbohydrate metabolism, as well as signal transduction and the process of apoptosis [157].

Other metabolic pathways not explicitly discussed in the reviewed studies may also play a role in MASLD progression. For example, miR-106a-5p influences MASLD via the NF-κB pathway, affecting inflammation [158]. MiR-33, along with *SREBP*-1 and *SREBP*-2, is upregulated by insulin resistance, contributing to MASLD [159]. miR-16 is involved in hepatic stellate cell activation and apoptosis of hepatic stellate cells by targeting *Bcl-2* as well as miR15b [160], while miR-200c-3p promotes fibrosis progression through Src kinase signaling [161]. Additionally, miR-20a-5p impacts liver glycogen synthesis and insulin signaling triggered by elevated glucose levels. It achieves this by targeting *p63*, which in turn regulates the expression of *p53* and *PTEN* [151]. Lastly, *MIR22HG* regulates p27^kip1^ through miR-24-3p, suppressing cancerous growth, which may be relevant to the progression of MASLD [162]. These pathways indicate a broader network of molecular processes involved in MASLD.

This review has some strength and limitations that may be mentioned. A key advantage of miRNAs is their notable stability in biological fluids, such as plasma and serum, which makes them ideal candidates as minimally invasive biomarkers for diagnosis, prognosis, and monitoring of MASLD. Additionally, this study follows the rigorous PRISMA guidelines for systematic reviews, ensuring a transparent, standardized, and reproducible selection and analysis process. The inclusion of a wide range of original human studies published in the last decade allows for an updated and comprehensive overview of circulating miRNAs associated with different MASLD stages. Furthermore, the focus on circulating miRNAs broadens the clinical applicability by highlighting non-invasive biomarkers that could complement or potentially replace more invasive liver biopsy techniques. Finally, the review identifies critical gaps and variability in current research, providing clear directions for future studies and emphasizing the need for standardization and validation.

This study has several limitations that should be acknowledged. First, the lack of standardization in miRNA extraction, quantification, and analysis methods may lead to inconsistencies in results between different studies, underscoring the urgent need for standardized protocols that facilitate their clinical application. Second, other limitation is the heterogeneity in miRNA expression, suggesting that these biomarkers may vary depending on the disease stage, the presence of comorbidities, and individual patient characteristics. In this regard, an important challenge will be the validation of these miRNAs in diverse clinical cohorts to ensure their reliability and robustness in broader populations. Furthermore, while initial research has demonstrated a strong association between certain miRNAs and the early stages of the disease, their predictive ability in advanced stages, such as cirrhosis or liver cancer, still needs to be more thoroughly evaluated. Third, a quantitative meta-analysis was not conducted due to the high heterogeneity among the included studies. Variability in miRNA detection methods, sample types, diagnostic criteria, and study populations limited direct comparability and could have compromised the validity of a pooled quantitative synthesis. While this decision was made to preserve methodological rigor, we recognize that a future meta-regression using more stringent inclusion criteria could offer valuable insights.

Fourth, the possibility of publication bias cannot be excluded, as studies reporting statistically significant or positive findings related to miRNA expression in MASLD are more likely to be published, which may overestimate the diagnostic or prognostic potential of these biomarkers. Moreover, there is considerable heterogeneity among the included studies regarding sample sizes, patient characteristics, disease stages, and biological sources (plasma, serum, liver tissue), further complicating direct comparison and interpretation of results.

In summary, miRNAs offer great potential in clinical practice for the diagnosis, prognosis, and treatment of MASLD. However, standardizing detection methods and continuing research to validate their utility across diverse populations and disease stages are essential.

## Conclusion

This review emphasizes the need to fill the gap in the knowledge of the role of miRNAs in MASLD and the potential use in its diagnosis, prognosis, and personalized treatment. Their stability in serum and plasma makes them ideal candidates for minimally invasive diagnostic tools, reducing the reliance on costly and invasive procedures like liver biopsies. Additionally, miRNAs can serve as key prognostic indicators, identifying patients at higher risk of disease progression, and guiding personalized treatment strategies. While further validation in diverse populations and stages of MASLD is necessary, the evolving understanding of miRNA biology suggests that they may soon play a role in the clinical management of this increasingly prevalent and burdensome condition. Continued research into the therapeutic modulation of miRNAs holds promise for developing targeted treatments that address the underlying mechanisms of MASLD, potentially improving patient outcomes and reducing healthcare costs.

## Electronic supplementary material

Below is the link to the electronic supplementary material.

Supplementary Material 1

Supplementary Material 2

## Data Availability

No datasets were generated or analysed during the current study.
