# Supplementary material for: The use of circulating miRNAs for the diagnosis, prognosis, and personalized treatment of MASLD
Source: J Physiol Biochem. 2025 Jul 16;81(3):589–609. doi: 10.1007/s13105-025-01110-w (PMC12373555; doi:10.1007/s13105-025-01110-w)
Supplement: Supplementary file 1 — Supplementary Material 1 [file 13105_2025_1110_MOESM1_ESM.pdf]

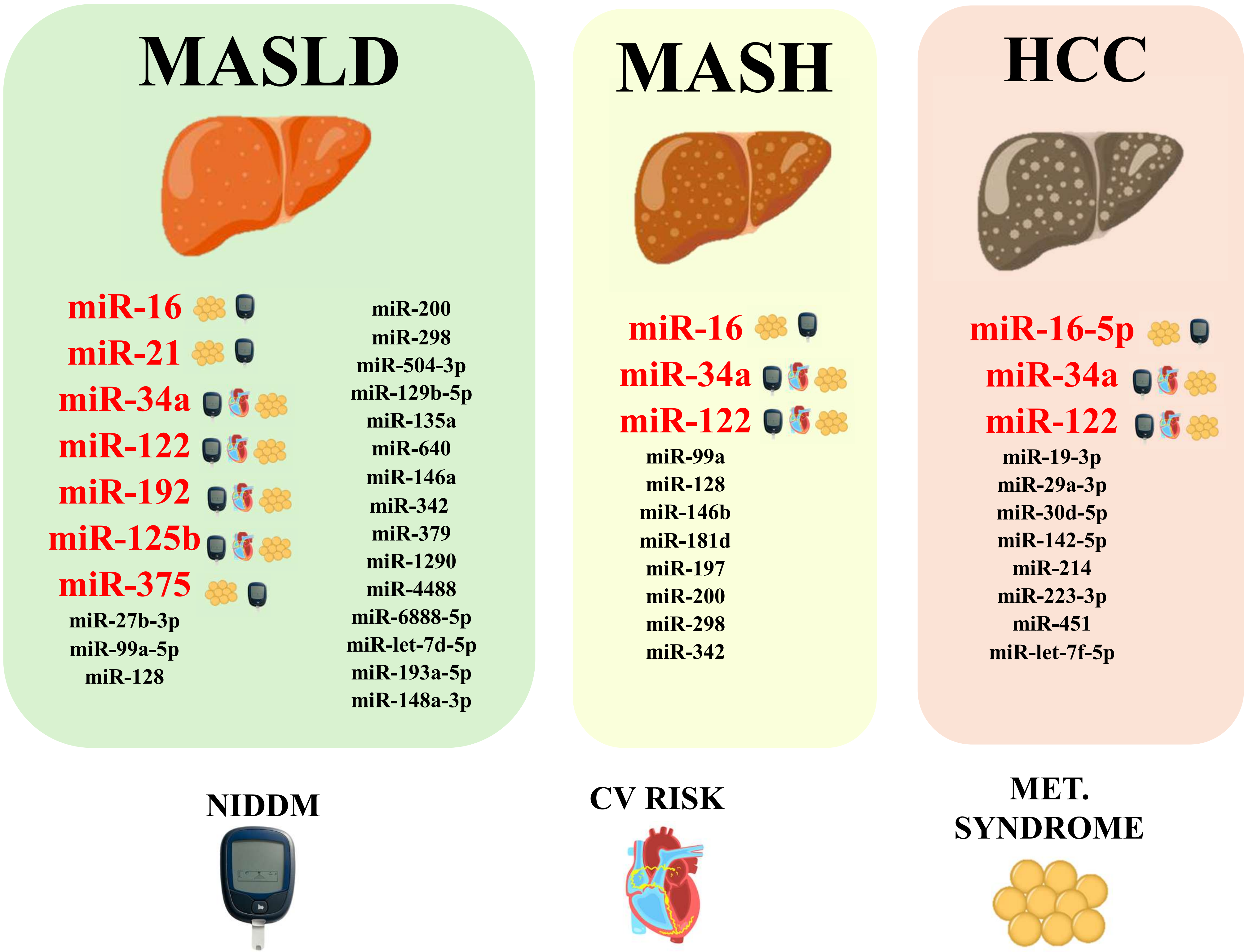
MASLD
miR-16
miR-21
miR-34a
miR-122
miR-192
miR-125b
miR-375
miR-27b-3p
miR-99a-5p
miR-128
miR-200
miR-298
miR-504-3p
miR-129b-5p
miR-135a
miR-640
miR-146a
miR-342
miR-379
miR-1290
miR-4488
miR-6888-5p
miR-let-7d-5p
miR-193a-5p
miR-148a-3p
MASH
miR-16
miR-34a
miR-122
miR-99a
miR-128
miR-146b
miR-181d
miR-197
miR-200
miR-298
miR-342
HCC
miR-16-5p
miR-34a
miR-122
miR-19-3p
miR-29a-3p
miR-30d-5p
miR-142-5p
miR-214
miR-223-3p
miR-451
miR-let-7f-5p
NIDDM
CV RISK
MET.
SYNDROME
