# Supplementary material for: The use of circulating miRNAs for the diagnosis, prognosis, and personalized treatment of MASLD
Source: J Physiol Biochem. 2025 Jul 16;81(3):589–609. doi: 10.1007/s13105-025-01110-w (PMC12373555; doi:10.1007/s13105-025-01110-w)
Supplement: Supplementary file 2 — Supplementary Material 2 [file 13105_2025_1110_MOESM2_ESM.docx]

The use of circulating miRNAs for the diagnosis, prognosis, and personalized treatment of MASLD.

*J Physiol Biochem*

Ana Luz Tobaruela-Resola^1^, Fermín I. Milagro^1,2,3*^, Paola Mogna-Pelaez^1^, María Jesús Moreno-Aliaga^,1,2,3^, Itziar Abete^1,2,3#^, M. Ángeles Zulet^1,2,3#^.

^1^Department of Nutrition, Food Sciences and Physiology and Centre for Nutrition Research, Faculty of Pharmacy and Nutrition, University of Navarra, 31008 Pamplona, Spain.

^2^Navarra Institute for Health Research (IdiSNA), 31008 Pamplona, Spain.

^3^ Centro de Investigación Biomédica en Red de Fisiopatología de la Obesidad y Nutrición (CIBERobn), Instituto de Salud Carlos III, 28029 Madrid, Spain.

#Contributed equally

*Authors to whom correspondence should be addressed.

* Fermín I. Milagro: Department of Nutrition, Food Science and Physiology, University of Navarra. C/ Irunlarrea 1, 31008. Pamplona, España. Tel: (+34)948425600. fmilagro@unav.es

**ORCID**

Ana Luz Tobaruela Resola, https://orcid.org/0009-0008-4484-8819

Fermín I. Milagro, https://orcid.org/0000-0002-3228-9916

Paola Mogna Pelaez, https://orcid.org/0000-0001-6058-312X

María Jesús Moreno Aliaga, https://orcid.org/0000-0002-2018-6434

Itziar Abete Goñi, https://orcid.org/0000-0002-6475-5387

María A. Zulet, <https://orcid.org/0000-0002-3926-0892>

Supplementary Table 1. Studies included in this systematic review.

| References | Country | Sample size | | | miRNAs investigated | | Sample sources | Biological process or metabolic pathway | | Stage of the disease | Function of miRNA in the study | | Age | Sex (Male) | | Sex (Female) |
| --- | --- | --- | --- | --- | --- | --- | --- | --- | --- | --- | --- | --- | --- | --- | --- | --- |
| Samy 2024[19] | AFRICA | 50 MASL patients, 50 MASH patients, and 50 healthy controls | | | miR-122; miR-128; miR-200; miR-298; miR-342 | | Pla**s**ma | LPS; TLR-4; FoxO3 | | MASL/  MASH | Diagnosis | | 40.85 (8.38) | 28 | | 22 |
| Elemeery 2019 [20] | AFRICA | 200 T2DM patients, 270 LC patients, 200 HCC patients, and 225 healthy controls | | | miR-34a; miR-221; miR-16; miR-23-3p; miR-122-5p; miR-198; miR-199a-3p | | Serum | Not reported | | HCC | Diagnosis | | 57.14 (8.43) | 591 | | 304 |
| Abdelgwad 2023 [21] | AFRICA | 55 controls and 55 MASLD patients | | | miR-29a | | Serum | Not reported | | MASH | Biological process and signaling pathways | | 43.2 | 18 | | 37 |
| Ragab 2023 [22] | AFRICA | 90 MASLD patients | | | miR-34a;  miR-192 | | Serum | Not reported | | MASLD | Diagnosis | | 40.08 (13.30) | 32 | | 58 |
| Albadawy 2021 [23] | AFRICA | 60 MASLD/MASH patients without suspicion of advanced fibrosis, 40 MASLD/MASH patients with suspicion of advanced fibrosis and 100 controls | | | miR-6888-5p | | Serum | Not reported | | MASLD/MASH | Diagnosis | | Not reported | 60 | | 31 |
| Mohamed 2021 [24] | AFRICA | 100 healthy individuals and 214 MASLD patients | | | miR-34 | | Serum | Not reported | | MASLD | Diagnosis | | 53.45 (13.65) | 158 | | 156 |
| Mohamed 2022 [25] | AFRICA | 157 patients with MASLD and 100 controls | | | miR-122 | | Serum | No reported | | MASLD/MASH | Diagnosis/Prognosis | | 47.25 | 143 | | 114 |
| Boonkaew 2023 [26] | ASIA | 70 MASLD patients and 35 healthy controls | | | miR-19-3p | | Plasma | Not reported | | NBNC-HCC (non-hepatitis C-related HCC) | Diagnosis/Prognosis | | 57.57 (11.11) | 88 | | 87 |
| Huang 2024 [27] | ASIA | 35 participants with non-steatosis, 35 with simple steatosis and 35 with MASH | | | miR-582-3p | | Plasma/ Fecal | TMBIM1 | | MASH | Biological process and signaling pathways | | Not reported | 42 | | 71 |
| Shen 2023 [28] | ASIA | 498 MASLD patients and 98 healthy controls | | | miR-4488 | | Serum | TNF; p53; RHGAP1; SLC10A1; SIX5 | | MASLD | Diagnosis | | 51.66 (15.58) | 46 | | 52 |
| Akuta 2022 [29] | ASIA | 81 MASLD patients | | | miR-122 | | Serum | Not reported | | MASLD | Prognosis | | 52-59 | 47 | | 34 |
| Kim 2021 [30] | ASIA | 12 MASLpatients and 12 MASH patients | | | miR-21-5p; miR-151a-3p; miR-192-5p; miR-4449 | | Serum | Not reported | | MASLD/MASH | Diagnosis | | 48.75 | 11 | | 13 |
| Muhammad 2020 [31] | ASIA | 52 MASH patients related liver cirrhosis (n=26 for each group of with and without HCC) | | | miR-182; miR-301a; miR-373 | | Serum | Not reported | | MASH | Prognosis | | 66 | 38 | | 14 |
| Okamoto 2020 [32] | ASIA | 80 MASLD patients and 10 controls | | | miR-379 | | Serum | IGF-1 | | MASLD | Diagnosis | | 48.6 (15.4) | 54 | | 25 |
| Akuta 2019 [33] | ASIA | 441 MASLD patients | | | miR-122 | | Serum | Not reported | | MASLD | Diagnosis | | 52 (20‐87) | 224 | | 175 |
| Ye 2018 [34] | ASIA | 15 T2DM patients without MASLD or 15 T2DM patients with MASLD | | | miR-17; miR-20a; miR-20b; miR-122; miR-126; miR-146; miR-130b; miR-375; miR-144; miR-192; miR-30d; miR-320a; miR-423 | | Plasma | Not reported | | MASLD | Diagnosis | | Not reported | Not reported | | Not reported |
| Zhang 2017 [35] | ASIA | 377 HCC patients | | | miR-363-5p | | Liver tissue | RGPD5; RGPD6; ZNF445; ZNF780B | | HCC | Prognosis | | Not reported | Not reported | | Not reported |
| Liu 2016 [36] | ASIA | 111 MASH patients | | | miR-122; miR-125b; miR-146b; miR-16; miR-21; miR-192; miR-27b; miR-34a | | Serum | Not reported | | MASH | Diagnosis | | 40.67 | 69 | | 42 |
| Akuta 2016 [37] | ASIA | 305 MASLD patients | | | miR-122 | | Serum | Not reported | | MASLD/  HCC | Diagnosis | | 51 (20-85) | 178 | | 127 |
| Ye 2015 [38] | ASIA | 19 normal liver patients, 10 steatosic liver and 16 MASH liver | | | miR-30e | | Liver tissue | MT1DP; MT1X; SRPRB; PDIA6; NANS; YIF1A; IGHG1; TCF4; VIM; PTGS2; PAN3; SRRM2 | | MASLD/MASH | Biological process and signaling pathways | | Not reported | 17 | | 26 |
| Miyaaki H 2014 [39] | ASIA | 67 MASLD patients | | | miR-122 | | Serum | Not reported | | MASLD | Prognosis | | 51.8 (17.4) | 27 | | 40 |
| Aghajanzadeh 2023 [40] | ASIA | | 70 MASLD patients | miR-146b; miR-194; miR-214 | | Plasma/ Serum | | | NEAT1; XIST; NF-κB; STAT3; TCF3; RELA; RUNX1 | MASLD | | Diagnosis | 46.8 (1.9) | | 24 | 15 |
| Ng 2023 [41] | ASIA | 141 colorectal polyp patients, of which 38 had MASLD | | | miR-18a/miR-16; miR-25-3p/miR-16; miR-18a/miR-21-5p; miR-18a/miR-92a-3p | | Serum | Not reported | | MASLD | Diagnosis | | 63.55 (9.26) | 79 | | 62 |
| Erdem 2023 [42] | ASIA | 180 MASLD patients and 60 healthy controls | | | miR-21; miR-197; miR-122 | | Serum | Not reported | | MASLD | Diagnosis | | 38.96 (10.37) | 105 | | 135 |
| Zhang 2022 [43] | ASIA | 7 MASLD patients and 5 controls | | | miR-200c-3p | | Serum | Not reported | | MASLD/  LSG (laparoscopic sleeve castrectomy) | Treatment | | 18 to 60 | Not reported | | Not reported |
| Zhang 2021 [44] | ASIA | 81 MASLD patients and 78 healthy patients | | | miR-20a-5p | | Serum | Not reported | | MASLD | Biological process and signaling pathways | | 56 (49.64) | 46 | | 35 |
| Zhang 2021 [45] | ASIA | 20 MASH liver tissue samples and 20 controls | | | miR-21 | | Liver tissue | SFRP5; PPARγ | | MASH | Biological process and signaling pathways | | 43.85 (3.89) | 24 | | 16 |
| Wang 2020 [46] | ASIA | 13 healthy controls and 14 MASLD patients | | | miR-20a-5p | | Plasma | CD36 | | MASLD | Biological process and signaling pathways | | 30–48 | 21 | | 6 |
| Hou 2021 [47] | ASIA | 40 MASLD patients and 40 healthy controls. | | | miR-223 | | Serum | Not reported | | MASLD | Treatment | | 45.3 (11.6) | 64 | | 16 |
| Zong 2020 [48] | ASIA | 67 MASLD and T2DM patients, 73 subjects with only MASLD, 68 subjects with only T2DM, and 66 healthy controls | | | miR-132 | | Serum | Not reported | | MASLD | Diagnosis | | 57.53 (6.59) | 189 | | 85 |
| Hu 2020 [49] | ASIA | 60 healthy controls, 60 MASLD patients and 120 AP patients | | | miR-192-5p | | Serum | Not reported | | MASLD/  ACUTE PANCREATITIS | Diagnosis | | 52.27 (11.28) | 138 | | 102 |
| Ando 2019 [50] | ASIA | 92 MASLD subjects and 383 controls | | | miR-20a; miR-27a; miR-126 | | Serum | Not reported | | MASLD | Diagnosis | | 63.5 (9.63) | 82 | | 293 |
| Huang 2019 [51] | ASIA | 30 MASLD patients and 30 healthy controls | | | miR-181a | | Serum | PPARα | | MASLD | Biological process and signaling pathways | | 50.95 | 10 | | 14 |
| Yamaura 2017 [52] | ASIA | 28 patients with HB (*n* = 6), HC (*n* = 4), PBC (*n* = 3), AIH (*n* = 3), MASH (*n* = 5), and DILI (*n* = 7) and 4 control patients | | | miR-345; miR-483-5p; miR-193b; miR-19a; miR-19b; miR-26b; miR-142-3p; miR-25; miR-451 | | Serum | Not reported | | MASH | Diagnosis | | 54.03 | 15 | | 13 |
| Yang 2017 [53] | ASIA | 11 patients with steatosis and 11 healthy control subjects | | | miR-423-5p | | Liver tissue | FAM3A; NFE2 | | MASLD | Biological process and signaling pathways | | 43.80 | 6 | | 6 |
| Ao 2016 [54] | ASIA | 58 mild MASLD and 47 moderate-severe MASLD patients; 53 MASH and 52 non-MASH patients; 50 healthy controls | | | miR-9 | | Liver tissue | Onecut2; SIRT1; REST; CoREST | | MASLD | Biological process and signaling pathways | | 46.35 (7.95) | 99 | | 56 |
| Salvoza 2016 [55] | ASIA | 36 healthy controls and 28 biopsy-proven MASLD patients. | | | miR-21; miR-34a; miR-122; miR-125b; miR-375 | | Serum | Not reported | | MASLD | Diagnosis | | 42.4 | 35 | | 29 |
| Sun 2015 [56] | ASIA | 25 MASLD patients and 12 healthy controls | | | miR-21 | | Serum | Not reported | | MASLD | Prognosis | | Not reported | Not reported | | Not reported |
| Xu 2015 [57] | ASIA | 50 MASLD patients and 30 healthy patients | | | miR-103 | | Serum | Not reported | | MASLD | Prognosis | | 50 (6.75) | 44 | | 36 |
| Celikbilek 2014 [58] | ASIA | 20 MASLD patients and 20 controls | | | miR-181d; miR-99a; miR-197; miR-146b | | Serum | Not reported | | MASLD | Diagnosis | | 43.63 (8.76) | 18 | | 22 |
| Tan 2014 [59] | ASIA | 20 patients and 20 controls | | | miR-122-5p; miR-1290; miR-27b-3p; miR-192-5p | | Serum | Not reported | | MASLD | Diagnosis | | 39.68 (7.21) | 31 | | 9 |
| Ebrahimpour-Koujan 2024 [60] | ASIA | 46 MASLD patients | | | miR-21; miR-122 | | Serum | Not reported | | MASLD | Treatment | | 45.60 (10.23) | 23 | | 23 |
| Wang 2023 [61] | ASIA | 5 MASLD patients and 5 controls | | | miR-34 | | Serum | Not reported | | MASLD | Prognosis | | 49.50 (8.20) | 3 | | 7 |
| Pervez 2022 [62] | ASIA | 100 patients with MASLD | | | miR-122; miR-21; miR-103a-2; miR-421; miR-375; miR-34a | | Plasma | Not reported | | MASLD | Treatment | | 47.7 | 58 | | 42 |
| Zhang 2020 [63] | ASIA | 8 MASLD patients and 8 control subjects | | | miR-223 | | Liver tissue | FOXO | | MASLD | Biological process and signaling pathways | | 47.66 | 58 | | 42 |
| Guo 2017 [64] | ASIA | 5 patients with biopsy-proven hepatic steatosis (5 of MASLD and 3 non-steatosis controls (2 of chronic hepatitis B (CHB), 1 of primary biliary cirrhosis (PBC), | | | miR-34a | | Liver tissue | Not reported | | MASLD | Biological process and signaling pathways | | 52.88 | 4 | | 4 |
| Wang 2017 [65] | ASIA | 25 patients with MASLD and 20 healthy controls | | | miR-181b | | Serum | Not reported | | MASLD | Biological process and signaling pathways | | Not reported | Not reported | | Not reported |
| Wang 2020 [66] | ASIA | 11 patients diagnosed with MASLD and 10 healthy controls | | | miR-30a-3p | | Serum | PPAR-α | | MASLD | Biological process and signaling pathways | | 51.15 | 17 | | 14 |
| Gim 2021 [67] | ASIA | 41 patients with MASLD | | | miR-let-7b-5p; miR-378h; miR-1184; miR-3613-3p; miR-877-5p; miR-602; miR-133b; miR-509-3p | | Venous bllood | No reported | | MASLD | Diagnosis/Prognosis | | 55 (43-63) | 13 | | 28 |
| Guney 2024 [68] | ASIA | 100 MASLD patients and 100 controls | | | miR-146a; miR-99a; miR-640 | | Venous blood | No reported | | MASLD | Diagnosis/Prognosis | | 47.87 | 80 | | 120 |
| Jiang 2021 [69] | ASIA | 50 patients with MASLD and 50 healthy individuals | | | miR-135a-3p; miR-129b-5p; miR-504-3p; miR-122 | | Serum | AMPK | | MASLD | Diagnosis | | 42.42 | 25 | | 75 |
| He 2019 [70] | ASIA | 67 individuals with MASLD and T2DM, 73 with MASLD but no T2DM, 68 individuals with T2DM but no MASLD, and 68 controls | | | miR-29b; miR-29a; miR-29c | | Peripheral blood samples | No reported | | MASLD | Diagnosis | | 57.55 | 191 | | 85 |
| Akuta 2016 [71] | ASIA | 36 HCC patients | | | miR-122 | | Serum | No reported | | HCC | diagnosis | | 54 | 20 | | 16 |
| Chen 2023 [72] | ASIA | 47 MASLD patients | | | miR30a-3 | | SERUM | ABCA1 | | MASLD | Treatment | | 458 | 32 | | 30 |
| Xhang 2019 [73] | ASIA | 25 MASLD/MASH and 19 control patients | | | miR-378 | | Liver tissue | *Ppargc1β* | | MASH | Treatment | | 44.73 | 31 | | 12 |
| Delik 2020 [74] | ASIA | 40 MASLD patients and 20 controls | | | miR-122 | | Serum | Not reported | | MASLD | diagnosis | | 39.89 | 30 | | 30 |
| Stoica 2024 [75] | EUROPE | 36 with MASLD and 12 controls | | | ΔmiR-33b+ miR-122, miR-192; miR-33a | | Plasma | Not reported | | MASLD | Diagnosis | | 52 | 14 | | 22 |
| Rodrigues 2023 [76] | EUROPE | 199 MASLD patients and 377 HCC patients (n = 366 HCC and n = 11 MASLD-HCC) | | | miR-21-5p | | Serum | PPARα | | MASH | Biological process and signaling pathways | | 47.57 | 66 | | 133 |
| Lima 2021 [77] | EUROPE | 108 subjects with MASLD | | | miR-21; miR-29a; miR-122; miR-155; miR-181a | | Plasma/ Serum | Not reported | | MASLD | Diagnosis | | 56.8 (9.4) | 23 | | 85 |
| Erhartova 2019 [78] | EUROPE | 116 liver transplant subjects | | | miR-33a | | Serum | Not reported | | MASLD/MASH | Diagnosis | | 56.8 | 60 | | 56 |
| Lambrecht 2019 [79] | EUROPE | 92 patients were diagnosed with no or minimal fibrosis (F0–1) and 116 patients with significant fibrosis (F ≥ 2) | | | miR-451a; miR-142-5p; miR-let-7f-5p; miR-378a-3p; miR-29a-3p; miR-122-5p | | Plasma | Not reported | | Liver fibrosis | Diagnosis | | 54.5 | 136 | | 72 |
| Soronen 2016 [80] | EUROPE | 15 MASLD patients and 15 the non‐MASLD patients | | | miR‐103a‐2; miR‐106b; miR‐576‐5p; miRPlus‐I137; miR‐892a; miR‐1282; miR‐3663‐5p; miR‐3924 | | Liver tissue | Not reported | | MASLD | Prognosis | | 47.85 | 10 | | 19 |
| Becker 2015 [81] | EUROPE | 137 MASLD patients and 61 healthy controls | | | miR-122; miR-192; miR-21 | | Serum | Not reported | | MASH | Prognosis | | 40.6 | 26 | | 73 |
| Rusu 2023 [82] | EUROPE | 14 participants with HCC asociated with MASLD and 41 participand with MASLD | | | miR-21-5p; miR-34a-5p; miR-130a-3p; miR-155-3p | | FFPE tissue | WNT; P-53 | | HCC/  MASLD | Diagnosis | | 59.7 | 34 | | 21 |
| Infante-Menéndez 2023 [83] | EUROPE | 21 normal liver histology and 30 liver steatosis patients | | | miR-let-7d-5p | | Liver tissue | AKT; IGF1; IGF1R; INSR | | MASLD | Diagnosis | | 51.23 (14.72) | 30 | | 31 |
| Vulf 2021 [84] | EUROPE | 60 patients | | | miR-195-5p; miR-16-5p; miR-374a-5p; miR-1-3p; miR-23a-3p; miR-423-5p; miR-143-5p; miR-200c-3 | | Serum | Not reported | | MASLD/MASH | Biological process and signaling pathways | | 37.7 (9.7) | 7 | | 9 |
| Auguet 2016 [85] | EUROPE | 62 patients with morbid obesity, 30 patients with moderate obesity and 8 patients with normal-weight | | | miR-122; miR-33b | | Liver tissue | Not reported | | MASLD/MASH | Diagnosis | | 47.47 (4.15) | Not reported | | 122 |
| Amerikanou 2021 [86] | EUROPE | 27 patients received Mastiha and 40 patients received placebo | | | miR-155 | | Plasma | Not reported | | MASLD | Treatment | | 49 | 47 | | 20 |
| Quintás 2022 [87] | EUROPE | 20 human livers from organ controls and 23 human sera from biopsy-proven MASLD patients | | | miR-10a-5p; miR-98-5p; miR-19a-3p; miR-30e-5p; miR-32-5p; miR-145-5p | | Serum/ Liver tissue | Not reported | | MASLD | Diagnosis | | 51(11) | 11 | | 12 |
| Soluyanova 2024 [88] | EUROPE | 20 MASLD patients and 24 patients with morbid obesity | | | miR-145-3p; miR-122-5p; miR-143-3p; miR-500a-5p; miR-182-5p | | Liver tissue/Serum | No reported | | MASLD | Prognosis | | 46 | 13 | | 33 |
| Tobaruela-Resola 2024 [89] | EUROPE | 55 MASLD patients | | | miR-122; miR-126-5p; miR15b-3p; miR29b-3p; miR151a-3p; miR-21-5p | | Serum | No reported | | **MASLD** | diagnosis | | 53,14 | 34 | | 21 |
| Tobaruela-Resola 2024 [90] | EUROPE | 55 MASLD patients | | | miR-122-5p, miR15b-3p | | Serum | No reported | | **MASLD** | diagnosis | | 53,14 | 34 | | 21 |
| Lendvai 2014 [91] | EUROPE | 18 CHC, 39 CHC-Steatosis and 18 steatosis patients | | | miR-21; miR-33a; miR-96; miR-122; miR-125b; miR-221; miR-224 | | FFPE tissue | No reported | | MASLD and CHC | diagnosis | | 42.8 | 36 | | 29 |
| Pillai 2020 [92] | NORTH AMERICA | 62 patients | | | miR-122; miR-34a; miR-375; miR-16; miR-21 | | Plasma | Not reported | | MASLD | Diagnosis | | 47.7 (2.9) | Not reported | | 62 |
| Ezaz 2020 [93] | NORTH AMERICA | 182 patients | | | miR-34a; miR-122; miR-192; miR-200a | | Serum | eLP-IR 3; I148M; TM6SF2; E167K | | MASLD | Diagnosis | | 50.6 | 112 | | 70 |
| Estep 2015 [94] | NORTH AMERICA | 24 patients with obesity undergoing bariatric surgery with biopsy-proven MASLD | | | miR-100; miR-99a; miR-99b; miR-125b; miR-let-7b; miR-let-7c; miR-26a | | Adipose tissue | IL13RA; mTOR; IL20; SEMA4C; FAS | | MASH | Diagnosis | | 45.95 | 6 | | 18 |
| Johnson 2021 [95] | NORTH AMERICA | 183 MASLD patients and 10 controls | | | miR-193a-5p; miR-378d; miR-378d | | Serum | Not reported | | MASLD | Diagnosis | | 55 (44–64) | 110 | | 73 |
| Newman 2022 [96] | NORTH AMERICA | 8 MASL patients, 6 biopsy-proven MASH and 14 healthy controls | | | miR-122; miR-192; miR-128-3p | | Plasma | Not reported | | MASLD | Diagnosis | | 49.5 (16.5) | 14 | | 14 |
| He 2019 [97] | NORTH AMERICA | 10 controls and 14 MASH patients | | | miR-223 | | Liver tissue | Cxcl10; Taz | | MASH | Biological process and signaling pathways | | 51.8 | 10 | | 14 |
| Mehta 2016 [98] | NORTH AMERICA | 32 patients with angiography-confirmed CAD to those with MASLD and 12 without CAD | | | miR-132; miR-143; miR-145; miR-211; miR-146a; miR-30c; miR-161; miR-241 | | Plasma | Not reported | | MASLD | Prognosis | | 62.50 (9.60) | 17 | | 27 |
| Zarrinpar 2016 [99] | NORTH AMERICA | 18 MASLD patients and 62 controls | | | miR-331-3p; miR-30c | | Serum | Not reported | | MASLD | Diagnosis | | 52.25 (3.05) | 18 | | 62 |
| Niture 2023 [100] | NORTH AMERICA | 40 HCC patients | | | miR-483-5p | | Liver tissue | Not reported | | HCC | Prognosis | | 60.6 | 31 | | 9 |
| Tran 2017 [101] | NORTH AMERICA | 10 Controls, 16 MASH non-fatty liver patients and 20 MASH fatty liver | | | miR-141; miR-200c | | Liver tissue | Not reported | | MASLD | Prognosis | | 57.55 | 13 | | 23 |
| Hanin 2018 [102] | NORTH AMERICA | 5 patients diagnosed with MASLD, 3 normal tissue and 2 NAT to cancer and benign conditions | | | miR-132 | | Liver tissue | Not reported | | MASLD | Biological process and signaling pathways | | 58.7 | 2 | | 8 |
| Xu 2015 [103] | NORTH AMERICA | 8 MASH patients | | | miR-34a | | Liver tissue | Not reported | | MASH | Biological process and signaling pathways | | 54.15 | Not reported | | Not reported |
| Jones 2018 [104] | NORTH AMERICA | 186 patients with HCC | | | miR-26a | | FFPE tissue | No reported | | HCC | Prognosis/Treatment | | 61.8 | 139.5 | | 46.5 |
| Dattaroy 2015 [105] | NORTH AMERICA | 3 NASH and 3 controls patients | | | miR21 | | Liver tissue | TGF-β, SMAD2/3-SMAD4 NF-κB | | MASH | diagnosis | | No reported | No reported | | No reported |
| Pirola 2015 [106] | SOUTH AMERICA | 48 patients | | | miR-122; miR-192; miR-19a; miR-19b; miR-125b; miR-375 | | Serum | Not reported | | MASLD | Diagnosis | | 49.47 | 137 | | 163 |
| Cansanção 2020 [107] | SOUTH AMERICA | 13 patients received n-3 PUFA, and 11 patients received control supplements | | | miR-122 | | Plasma | Not reported | | MASLD | Treatment | | 58.5 | 17 | | 7 |
| Hendy 2019 [108] | SOUTH AMERICA | 124 patients of simple steatosis and 86 of MASH | | | miR-122; miR-34a; miR-99a | | Serum | Not reported | | MASH/  MASLD | Diagnosis | | 49.47 (7.54) | 24 | | 27 |

Abbreviations: MASLD, Metabolic Dysfunction-Associated Steatotic Liver Disease; MASH, Metabolic Associated Steatohepatitis; HCC, Hepatocellular Carcinoma; PUFA, Polyunsaturated Fatty Acid; NBNC-HCC, non-hepatitis C-related HCC; miRNA, microRNA; CHB, Chronic Hepatitis B; CHC, Chronic Hepatitis C; PBC, Primary Biliary Cirrhosis; LSG Laparoscopic Sleeve Gastrectomy; T2DM, Type 2 Diabetes Mellitus; CAD, Coronary Artery Disease; DILI, drug-induced liver injury; HB, Hepatitis B; HC, Hepatitis C; AIH, Autoinmune Hepatitis; LC, Liver Cirrhosis; NAT, Normal Adjacent Tissues; FFPE, Formalin-Fixed Paraffin-Embedded.
